# Supplementary material for: NAC blocks Cystatin C amyloid complex aggregation in a cell system and in skin of HCCAA patients
Source: Nat Commun. 2021 Mar 23;12:1827. doi: 10.1038/s41467-021-22120-4 (PMC7988011; doi:10.1038/s41467-021-22120-4)

Supplementary Table 1: Primers for cloning and mutagenesis

Primer Name	Sequence
CST3-myc Cloning Forward	5’-GATCGAATTCGCCACCATGGCCGGGCCCCTGCGCG-3’
CST3-myc Cloning Reverse	5’-TCGCGGCCGCCTACAGATCCTCTTCTGAGATGAGTTTTTGTTCG GCGTCCTGACAGGTGGATTTCG-3’
L68Q Mutagenesis Forward	5’-GTGAACTACTTCTTGGACGTCGAGCAGGGCCGAACCACGTGTACC-3’
L68Q Mutagenesis reverse	5’-GGTACACGTGGTTCGGCCCTGCTCGACGTCCAAGAAGTAGTTCAC-3’

Supplementary Figure 1

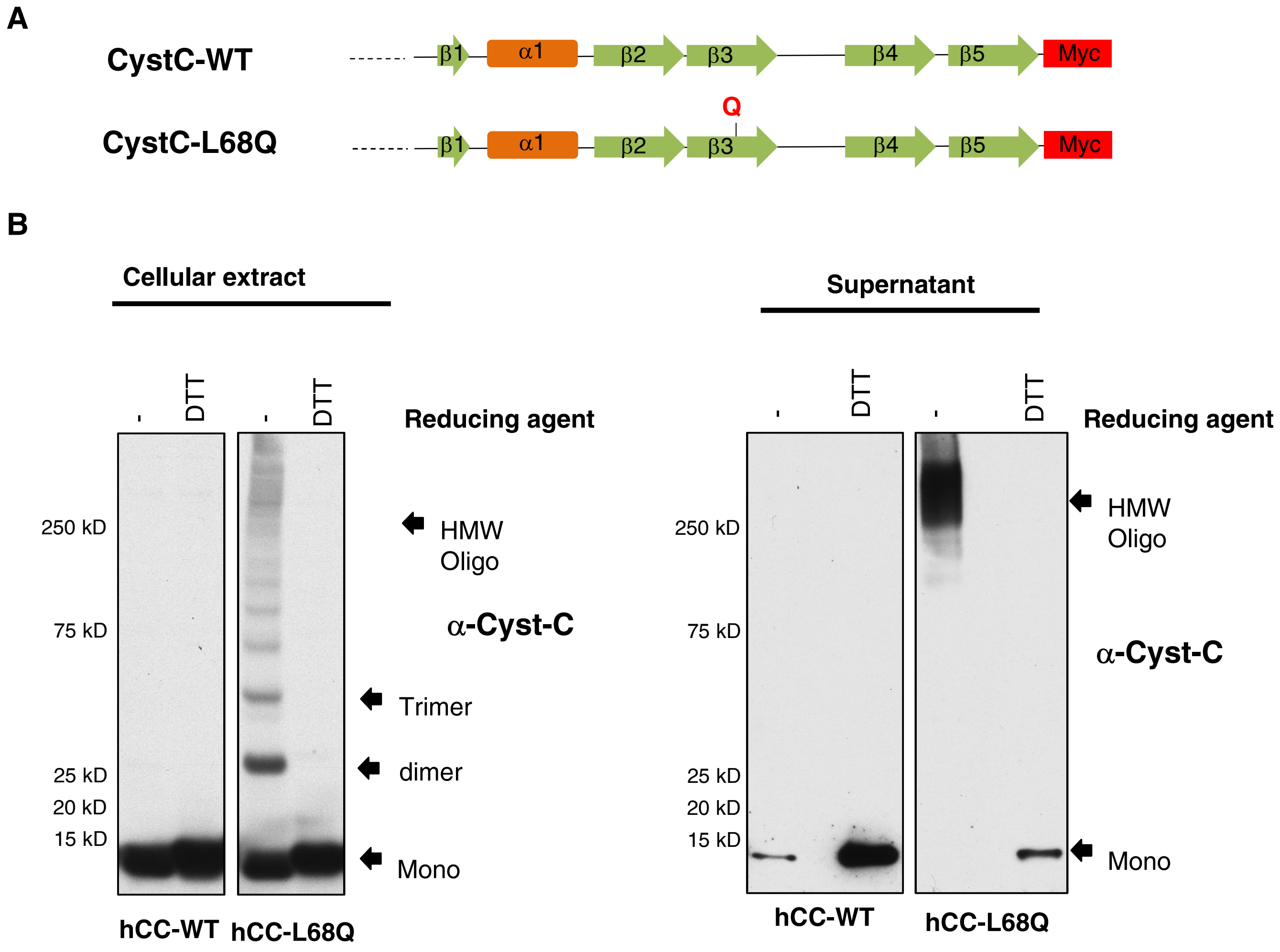

Genetically engineeredHEK-293T cells produce and secrete detectable levels of hCC (WT or L68Q) capable of oligomerizing under non-reducing conditions.

A. Schematic representation of WT and L68Q mutant hCC proteins. Dashed line represents the N-terminal signal peptide subject to proteolysis. The green arrows represent beta sheets, the orange rectangles represent alpha helices, and the red rectangle represents the Myc tag added to the C-terminal end.

B. (left panel) Lysates from HEK-293T cells stably expressing hCC WT or L68Q mutant or supernatants (right panel) were mixed with 2% SDS with or without the reducing agent DTT when indicated. Samples were subject to electrophoresis and CST3 levels examined by the Western blot procedure using anti-cystatin C antibody.

Suppplementary Figure 2 – biological replicates used in quantitation for Figure 1

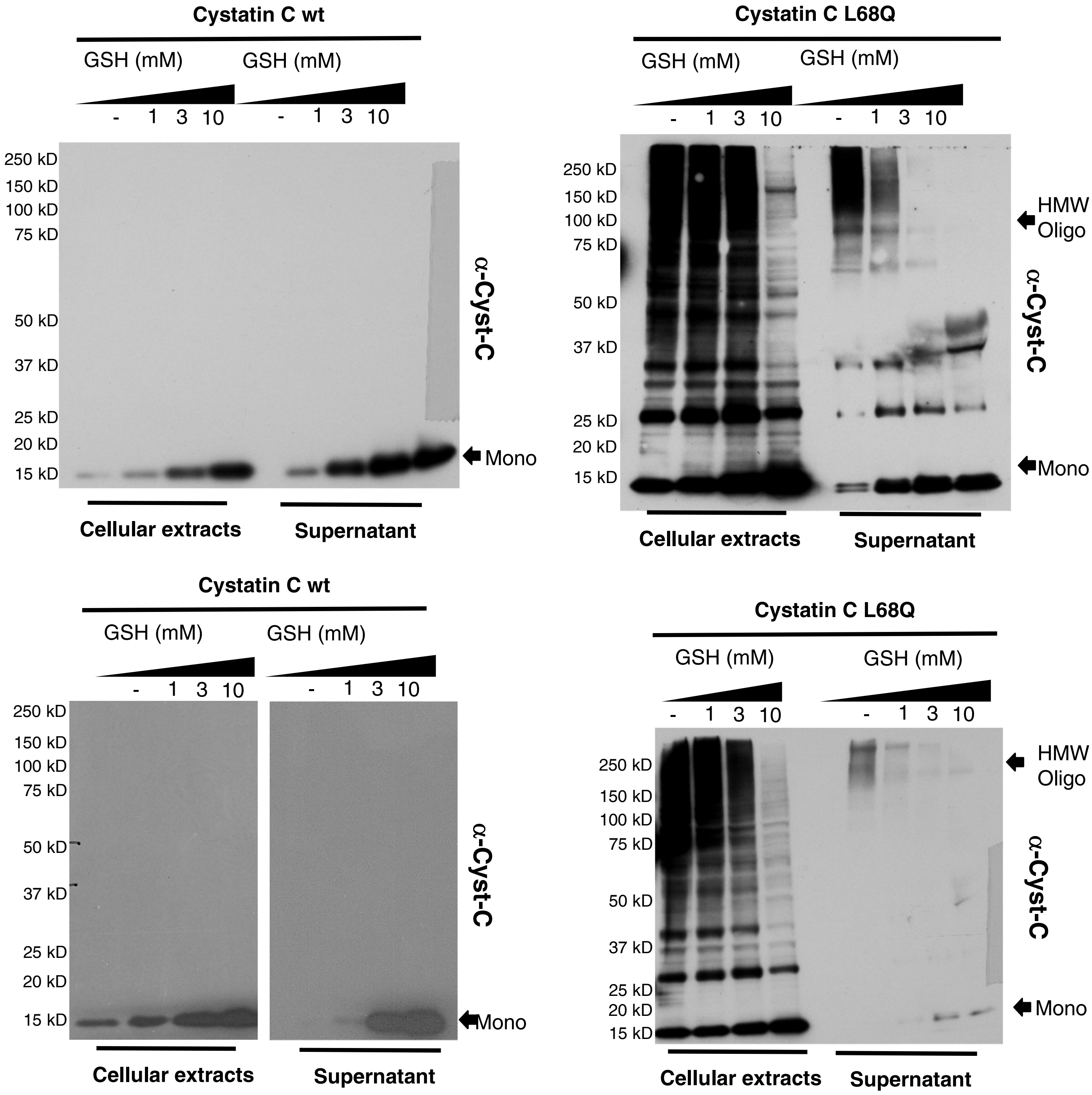

<u>Replicate experiments of Figure 1. Used to perform quantitation.</u>

Supplementary Figure 3 - biological replicates for Figure 2
Supplemental Figure 3A - Replicate 1

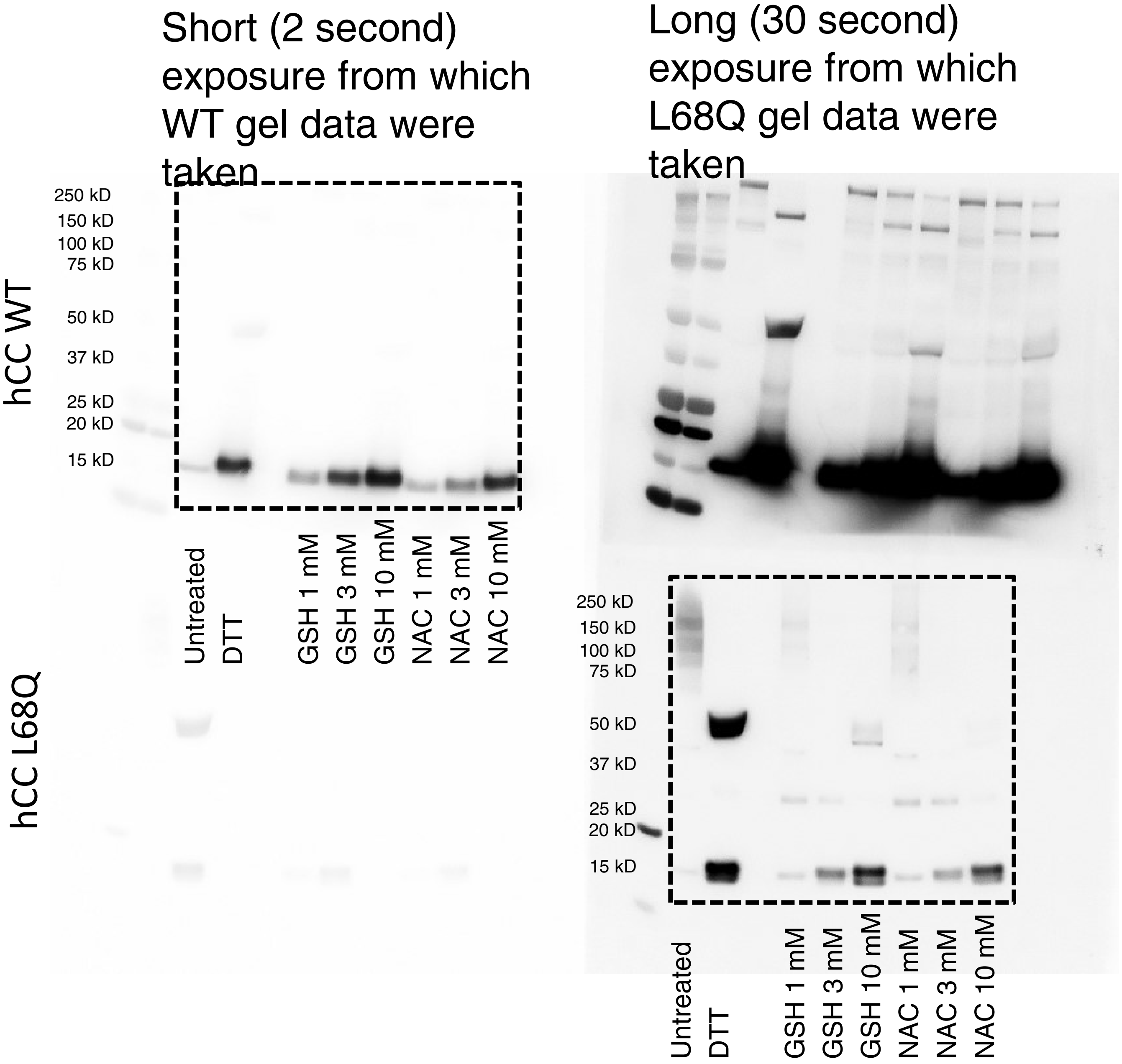

Supplemental figures 3A, 3B, and 3C are replicate experiments of Figure 2.
Used to perform quantitation. hCC-WT quantitation was performed using shorter exposures (top left). Due to the greater secretion of WT protein, the longer exposure (top right) is clearly overexposed. hCC-L68Q quantitation was performed using longer exposures (bottom right). Due to the lower secretion of mutant protein, it was barely detectable in shorter exposures (bottom left).

Supplementary Figure 3B - Replicate 2

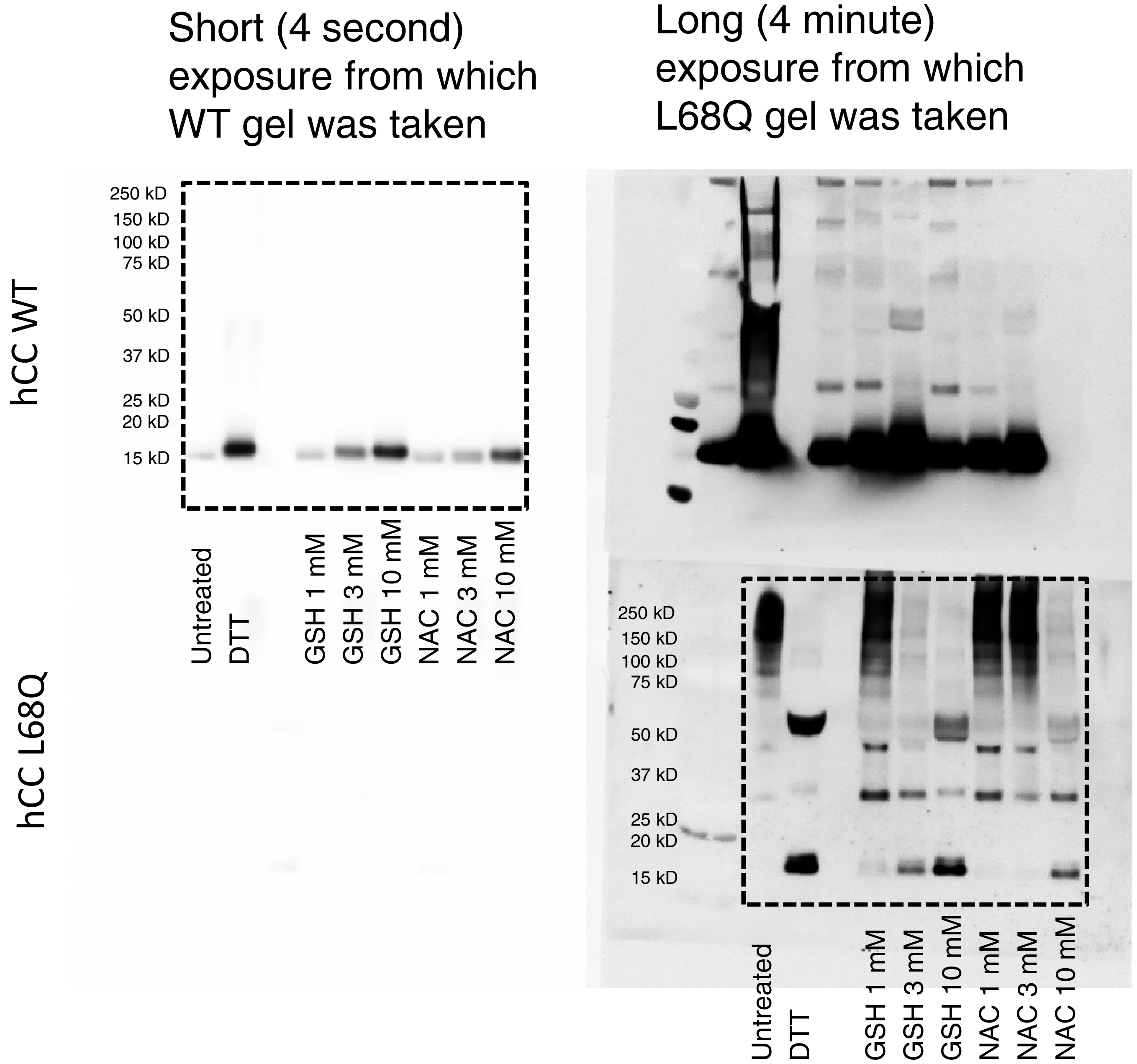

Supplementary Figure 3C - Replicate 3

These are the images from which the figure in the main text was made.

Short (4 second) exposure from which WT gel was taken

Long (4 minute) exposure from which L68Q gel was taken

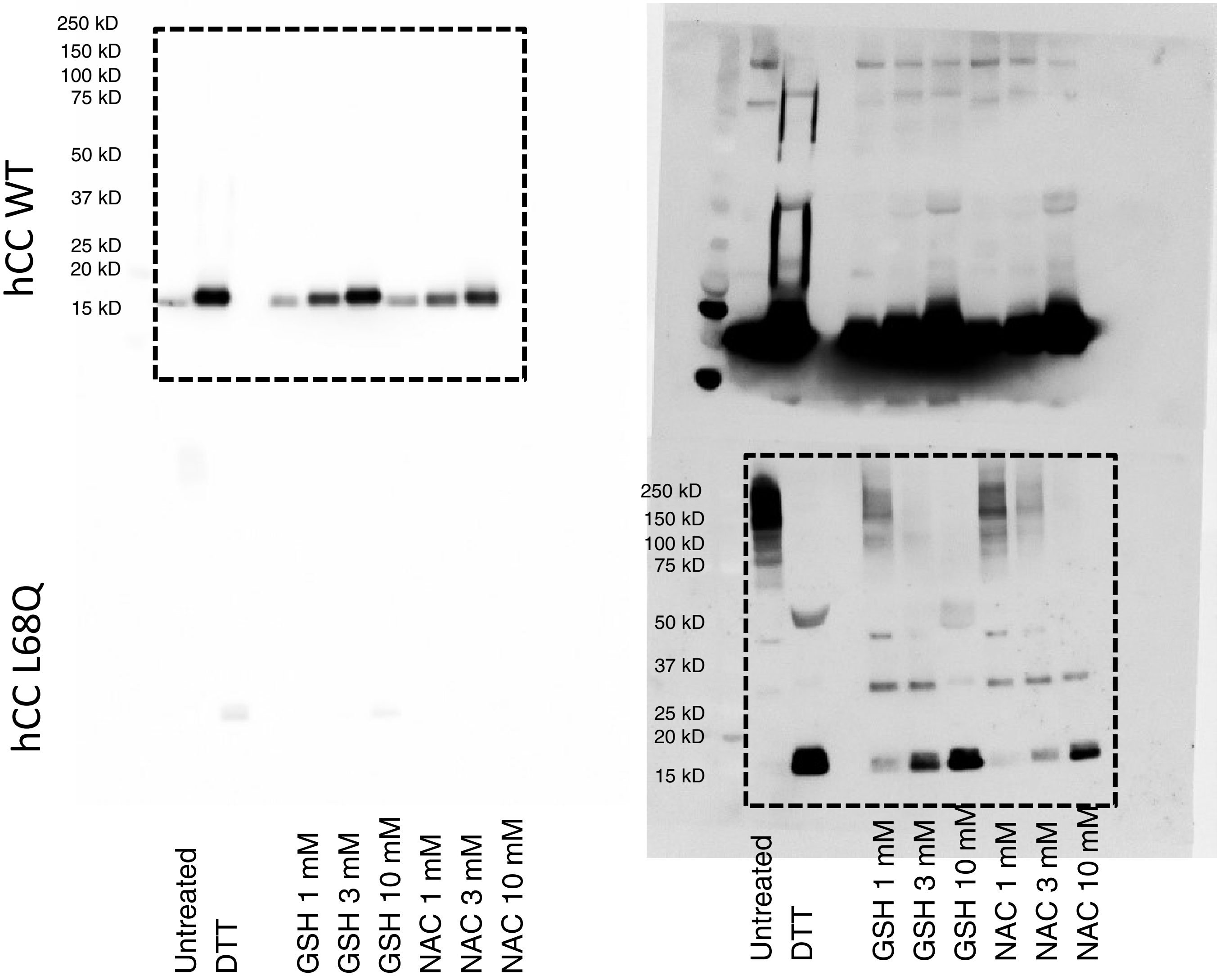

Supplementary figure 4 - biological replicates used in quantitation of figure 4

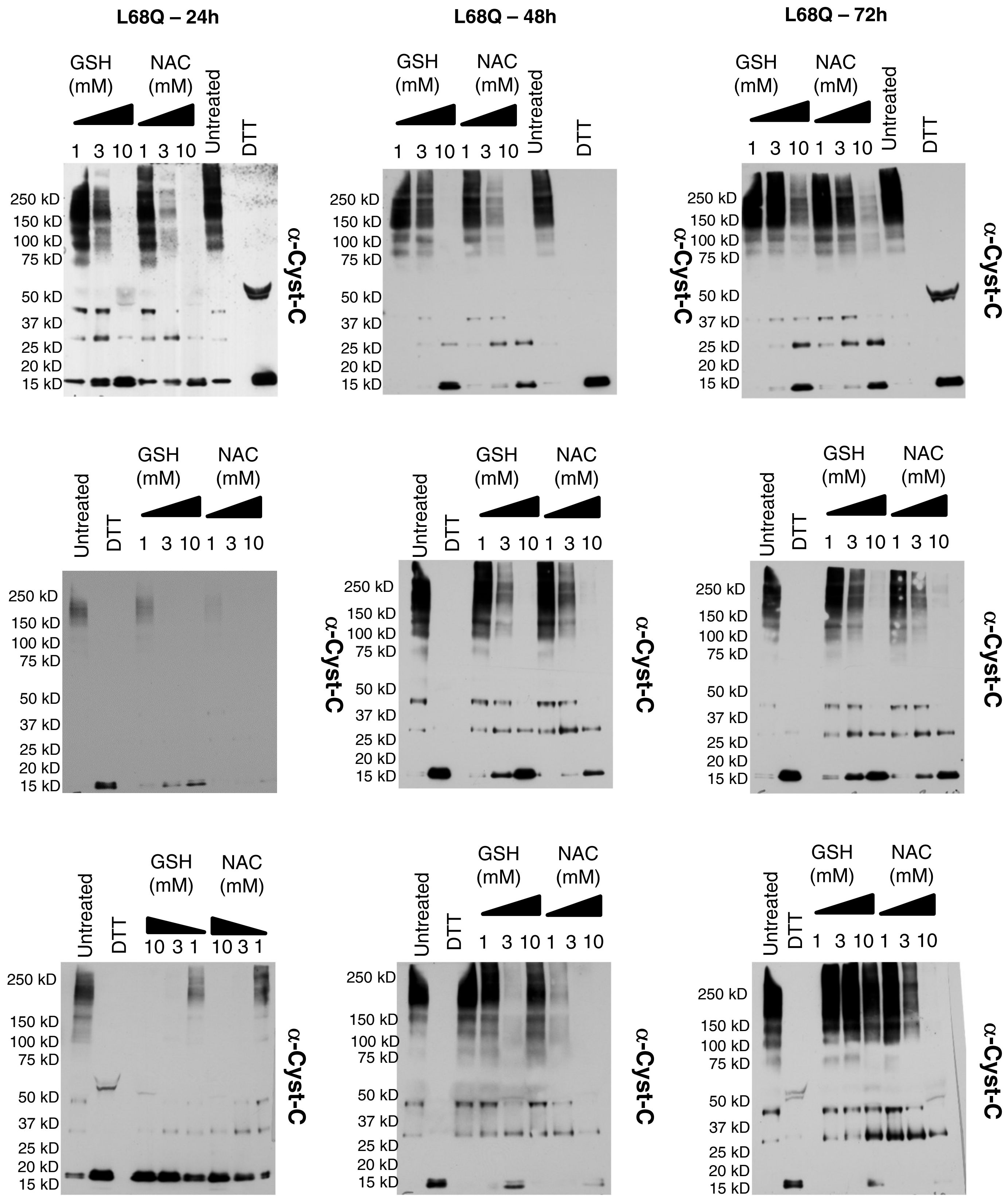

Supplementary figure 5 – biological replicates used for quantitation of figure 5

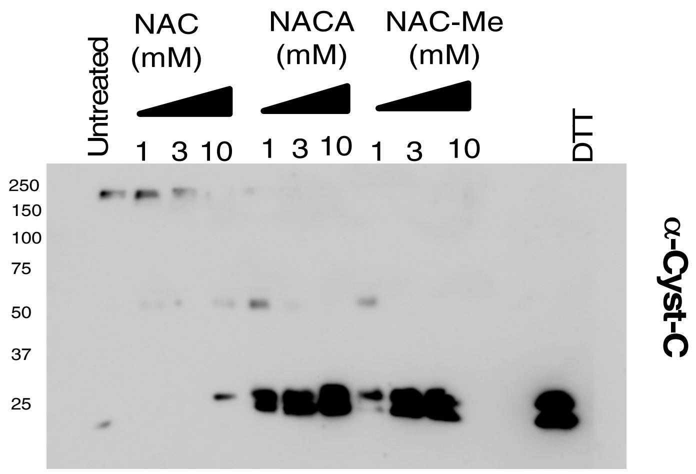

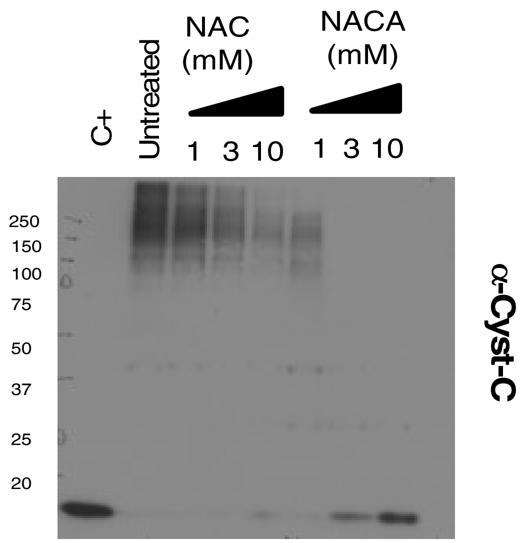

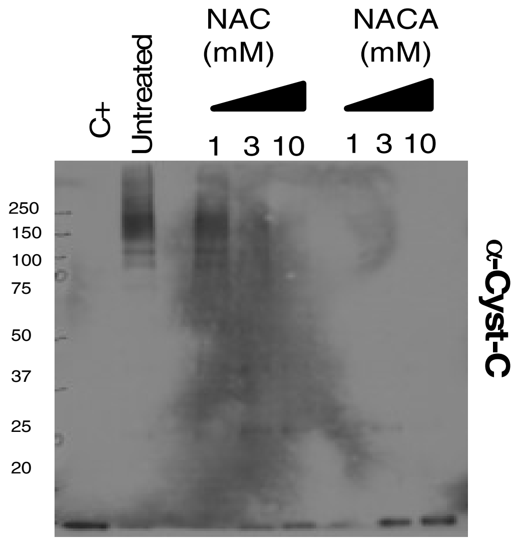

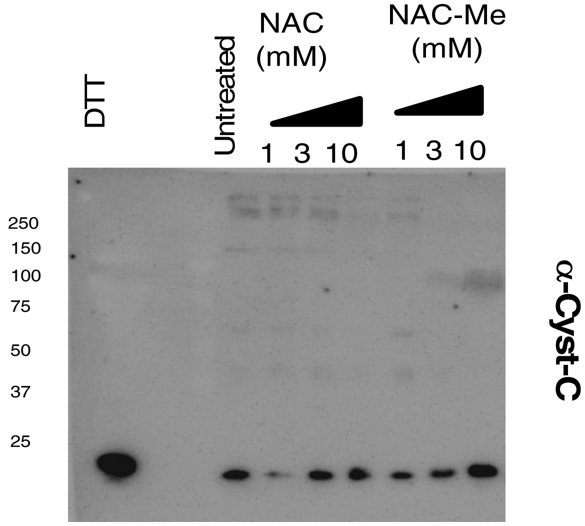

Supplementary Figure 6

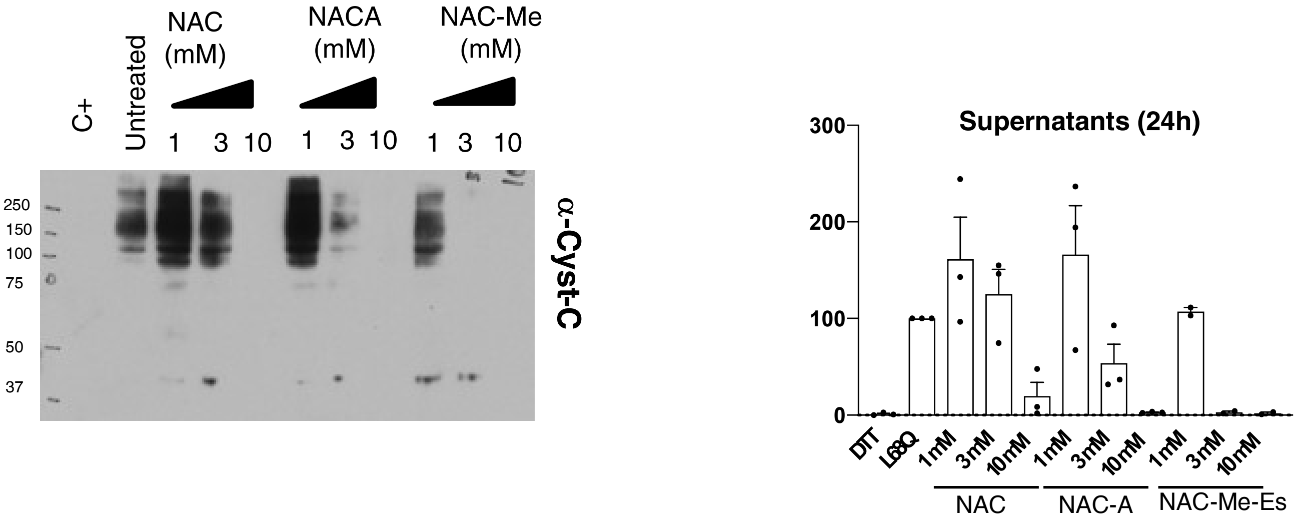

Supplemental Figure 6: 24 hour incubation with NAC derivatives impairs oligomerization of secreted hCC L68Q

Supernatants were incubated in the presence of the indicated concentrations of either NAC, NAC-amide (NACA) or NAC-Methyl esther (NAC-Me) for 24 hours. Samples were mixed with 2% SDS without reducing agents prior to electrophoresis, protein levels were detected by anti-cystatin C antibody, and amounts of high molecular weight hCC species (HMW) were quantitated (N=3 independent transfections). The histogram represents the quantification by densitometry of the Western blot bands for the high molecular weight fraction (HMW) relative to the untreated sample. Bars are means, error bars represent standard deviation. Bars are means, error bars represent standard deviation.

Supplementary figure 7 – Uncropped gels for images in Figure 3
Boxed areas are the regions presented in Figure 3

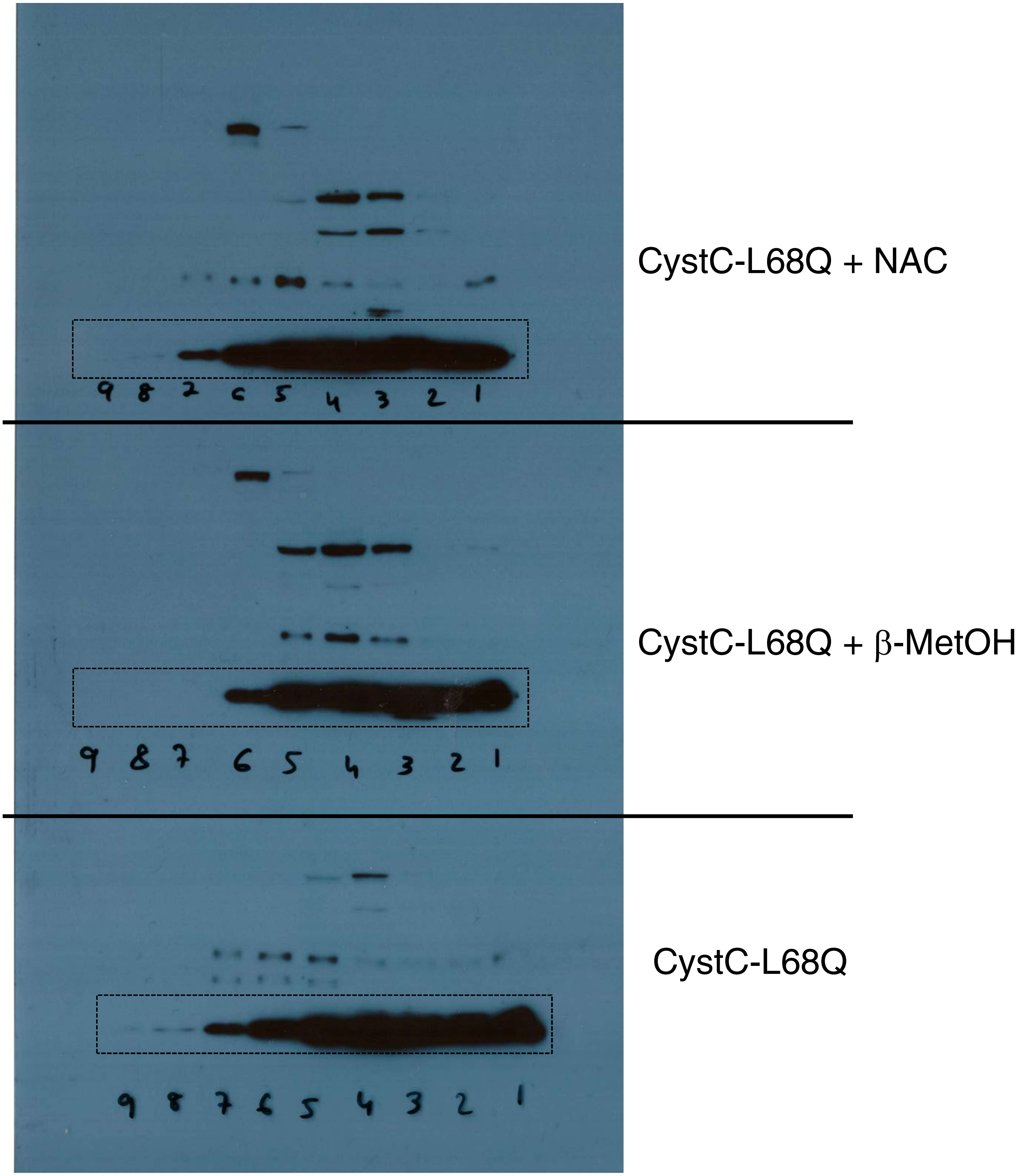

Supplement: Supplementary file 1 — Supplemenary Information [file 41467_2021_22120_MOESM1_ESM.pdf]
